# Draft genome sequence of *Ferrimicrobium acidiphilum* strain YE2023, isolated from a pulp of bioleach reactor

**DOI:** 10.1128/mra.00895-24

**Published:** 2024-11-12

**Authors:** Yulia A. Elkina, Aleksandr G. Bulaev, Alexey V. Beletsky, Andrey V. Mardanov

**Affiliations:** 1 Winogradsky Institute of Microbiology, Research Center of Biotechnology of the Russian Academy of Sciences, Moscow, Russia; 2 Institute of Bioengineering, Research Center of Biotechnology of the Russian Academy of Sciences, Moscow, Russia; Portland State University, Portland, Oregon, USA

**Keywords:** acidophilic microorganisms, extremophiles, iron-oxidizing microorganisms

## Abstract

We report the draft genome sequence of *Ferrimicrobium acidiphilum* strain YE2023, isolated from a pulp of a laboratory-scale bioleach reactor. The genome is 3,221,954 Mbp long with a guanine-cytosine content of 58.16%.

## ANNOUNCEMENT

*Ferrimicrobium acidiphilum* is a facultatively aerobic, mesophilic, extremely acidophilic, rod-shaped Gram-positive actinobacteria oxidizing ferrous iron ions and utilizing organic nutrients (1). Bacteria of the genus *Ferrimicrobium* have been detected in a microbial population of laboratory-scale reactors and columns (2, 3). Strain YE2023 was isolated from the acidic pulp of a laboratory-scale bioleach reactor (2). In the reactor, copper-nickel concentrate of low-grade ore of Nud II deposit (67.900104, 32.936505) (Kola Peninsula) collected in July 2019 was bioleached at 30°C in a batch mode (2). According to the analysis of the microbial population of the reactor by metabarcoding of V3-V4 fragments of 16S rRNA gene, the fraction of the sequences belonging to the genus *Ferrimicrobium* was about 1% (2). Strain YE2023 was isolated by 10-fold dilutions using 9KS medium containing ferrous sulfate (9.82 g/L) and yeast extract (YE) (0.02% wt/vol) with a pH of 1.8 (4) at 35°C. Pure culture obtained was presented by short rods. Purity was evaluated by uniform morphology of the cells. Pure culture was grown in the same medium lacking ferrous sulfate to check the presence of possible associated microorganisms. Also, culture purity was checked by analyzing the whole-genome sequence assembly. The strain has 99.22% identity to *Ferrimicrobium acidiphilum* strain T23 (accession no. NR_041798.1) by 16S rRNA gene sequence (GenBank database accession no. PQ460260). As the only strain of the genus was previously described (1), we performed sequencing of the genome of the strain YE2023.

The strain was deposited in Collection of Unique and Extremophilic Microorganisms of the Research Center of Biotechnology of the Russian Academy of Science (Moscow, Russia) under deposition number UQM 41822, 21 March 2024. To obtain genomic DNA, the strain was grown at 35°С in a shake flask with 100 mL of 9KS liquid medium (4). The cells were collected by centrifugation at 10,000 × *g*, and the biomass was washed twice with the medium lacking the ferrous sulfate and YE. Genomic DNA was extracted using PowerSoil Pro Kit (Qiagen, Germany). The genomic library was prepared using NEBNext Ultra II DNA Library Prep Kit (NEB, UK) according to the manufacturer’s recommendation. Sequencing was carried out using an Illumina MiSeq sequencer (Illumina, USA) producing 906,741 read pairs of 545,858,082 nucleotides; the average read length was 301 bp. Adapter sequences were removed using Cutadapt v.1.8 software (5); low-quality read ends were trimmed using Sickle v.1.33 with a quality threshold parameter *q* = 20 (6).

The processed reads were assembled *de novo* using SPAdes v.3.15.4 software (7) with “--isolate” parameter. The assembly produced 560 contigs with an average coverage of 99 reads, total genome size of 3,221,954 bp, an N50 value of 42,438 bp, and a guanine-cytosine content of 58.16%.

The genome completeness is 98.72%, estimated by CheckM2 v.1.0.1 (8). Annotation of the genome sequence performed using the National Center for Biotechnology Information Prokaryotic Annotation Pipeline (9) revealed 3,176 genes. Among these, 3,070 were identified as protein coding sequences; 47 were identified as tRNA genes; 3 were identified as complete rRNAs (5S, 16S, and 23S); and 51 were identified as pseudogenes. All tools were run with default parameters unless otherwise specified.

## Data Availability

Sequence of 16S rRNA gene has been deposited in the GenBank database under accession no. PQ460260. Sequence data associated with the annotated genome have been deposited in the National Center for Biotechnology Information under BioProject accession no. PRJNA1141276. This whole-genome shotgun project has been deposited at DDBJ/ENA/GenBank under the accession no. JBFSHR000000000. The version described in this paper is version JBFSHR000000000. The Illumina reads can be found under Sequence Read Archive (SRA) accession no. SRA–SRR30017611.
